# Investigation of spinal cerebrospinal fluid-contacting neurons expressing PKD2L1: evidence for a conserved system from fish to primates

**DOI:** 10.3389/fnana.2014.00026

**Published:** 2014-05-06

**Authors:** Lydia Djenoune, Hanen Khabou, Fanny Joubert, Feng B. Quan, Sophie Nunes Figueiredo, Laurence Bodineau, Filippo Del Bene, Céline Burcklé, Hervé Tostivint, Claire Wyart

**Affiliations:** ^1^Institut du Cerveau et de la Moelle Épinière, Hôpital de la Pitié-SalpêtrièreParis, France; ^2^Institut National de la Santé et de la Recherche Médicale UMR 1127Paris, France; ^3^Centre National de la Recherche Scientifique UMR 7225Paris, France; ^4^UPMC Univ. Paris 06Paris, France; ^5^Muséum National d'Histoire NaturelleParis, France; ^6^Centre National de la Recherche Scientifique UMR 7221Paris, France; ^7^Institut National de la Santé et de la Recherche Médicale UMR S 1158Paris, France; ^8^Institut CurieParis, France; ^9^Centre National de la Recherche Scientifique UMR 3215Paris, France; ^10^Institut National de la Santé et de la Recherche Médicale U 934Paris, France

**Keywords:** cerebrospinal fluid-contacting neurons (CSF-cNs), PKD2L1, mouse, macaque, zebrafish, GABAergic neurons, spinal cord

## Abstract

Over 90 years ago, Kolmer and Agduhr identified spinal cerebrospinal fluid-contacting neurons (CSF-cNs) based on their morphology and location within the spinal cord. In more than 200 vertebrate species, they observed ciliated neurons around the central canal that extended a brush of microvilli into the cerebrospinal fluid (CSF). Although their morphology is suggestive of a primitive sensory cell, their function within the vertebrate spinal cord remains unknown. The identification of specific molecular markers for these neurons in vertebrates would benefit the investigation of their physiological roles. PKD2L1, a transient receptor potential channel that could play a role as a sensory receptor, has been found in cells contacting the central canal in mouse. In this study, we demonstrate that PKD2L1 is a specific marker for CSF-cNs in the spinal cord of mouse (*Mus musculus*), macaque (*Macaca fascicularis*) and zebrafish (*Danio rerio*). In these species, the somata of spinal PKD2L1^+^ CSF-cNs were located below or within the ependymal layer and extended an apical bulbous extension into the central canal. We found GABAergic PKD2L1-expressing CSF-cNs in all three species. We took advantage of the zebrafish embryo for its transparency and rapid development to identify the progenitor domains from which *pkd2l1*^+^ CSF-cNs originate. *pkd2l1*^+^ CSF-cNs were all GABAergic and organized in two rows—one ventral and one dorsal to the central canal. Their location and marker expression is consistent with previously described Kolmer–Agduhr cells. Accordingly, *pkd2l1*^+^ CSF-cNs were derived from the progenitor domains p3 and pMN defined by the expression of *nkx2.2a* and *olig2* transcription factors, respectively. Altogether our results suggest that a system of CSF-cNs expressing the PKD2L1 channel is conserved in the spinal cord across bony vertebrate species.

## Introduction

Spinal cerebrospinal fluid-contacting neurons (CSF-cNs) were identified and described by Kolmer (1921, 1931) and Agduhr (1922) in over 200 species based on cellular morphology, location, and Nissl staining. They independently noted that these cells exhibit an apical bulbous extension in the central canal and send basal axonal projections to other cells. Their observations suggested that CSF-cNs could constitute a sensory organ (referred to as the sagittal organ by Kolmer) interfacing the cerebrospinal fluid (CSF) with the nervous system at the level of the spinal cord.

Since the discovery of CSF-cNs, electron microscopy studies have shown that these cells exhibit a sensory tuft, previously referred to as a “brush border” (Dale et al., 1987b), a “central body” (Vigh and Vigh-Teichmann, 1973) or a “bud” (Stoeckel et al., 2003). The apical bulbous extension projects from the perikarya of CSF-cNs toward the central canal and ends with a terminal bud in contact with its lumen. This apical extension is characterized by the dendritic marker microtubule-associated protein 2 (MAP2) (Orts-Del'Immagine et al., 2014).

Immunohistochemistry (IHC) studies showed evidence for GABAergic CSF-cNs in many species such as rat (Barber et al., 1982; Stoeckel et al., 2003), turtle (Reali et al., 2011), African clawed frog (Dale et al., 1987a; Binor and Heathcote, 2001), zebrafish (Bernhardt et al., 1992; Martin et al., 1998; Higashijima et al., 2004a,b; Wyart et al., 2009; Yang et al., 2010), eel and trout (Roberts et al., 1995), dogfish (Sueiro et al., 2004), and lampreys (Brodin et al., 1990; Christenson et al., 1991a,b; Melendez-Ferro et al., 2003; Robertson et al., 2007; Rodicio et al., 2008; Villar-Cervino et al., 2008). In African clawed frog and zebrafish, these GABAergic CSF-cNs were named Kolmer–Agduhr cells (KAs) to distinguish them from ciliated ependymal cells (Dale et al., 1987a; Bernhardt et al., 1992). In these species CSF-cNs project an ascending axon ventrally in the spinal cord (Dale et al., 1987a; Bernhardt et al., 1992; Higashijima et al., 2004a; Wyart et al., 2009).

Despite the anatomical studies of CSF-cNs and their implication in modulating locomotion (Wyart et al., 2009), their physiological role in vertebrates remains unknown. One obstacle to answering this question is the lack of a specific genetic marker to identify these cells. Recently the calcium-permeable polycystic kidney disease 2-like 1 (PKD2L1) channel (Huang et al., 2006; Ishimaru et al., 2006), first identified in kidney, retina, and heart (Basora et al., 2002) was found to be expressed in cells contacting the CSF at the level of the brainstem and spinal cord in mouse (Huang et al., 2006; Orts-Del'Immagine et al., 2012). This channel belongs to the family of Transient Receptor Potential (TRP) channels. These are known to be chemo-, thermo- or mechano-sensitive (Delmas, 2004; Delmas et al., 2004). Expression of this channel and location of these cells at the interface with the CSF are consistent with the hypothesis that CSF-cNs have a proprioceptive sensory function.

In this study, we investigated whether PKD2L1 was a specific marker for CSF-cNs across vertebrates, by examining mouse, macaque and zebrafish. In mouse and macaque PKD2L1 was enriched in the sensory tuft of CSF-cNs in cervical, thoracic and lumbar spinal cord. The soma of PDK2L1^+^ cells was located in the ependymal layer or just underneath. In both species, a significant proportion of PKD2L1^+^ CSF-cNs was distinctly identified as GABAergic. We took advantage of the zebrafish model organism to thoroughly characterize the properties and lineage of *pkd2l1*^+^ cells in the spinal cord. In this model, all *pkd2l1*^+^ cells contacting the central canal were confirmed as GABAergic neurons. Reciprocally, all KAs, defined by their location and their GABAergic phenotype expressed *pkd2l1*. Using specific transgenic lines, we confirmed that these cells derive from two progenitor domains. The dorsal *pkd2l1*^+^ KAs originated from pMN and were labeled by the Enhanced Green Fluorescent Protein (EGFP) in the *Tg(olig2:EGFP)* while the ventral *pkd2l1*^+^ KAs originated from p3 and were labeled by *Tg(nkx2.2a:mEGFP)*. Altogether our data show that PKD2L1 is a specific marker of CSF-cNs shared between multiple vertebrate species. We found evidence for PKD2L1-expressing GABAergic CSF-cNs in all examined species. Our results on the developmental origin of CSF-cNs in zebrafish open new paths of investigation in mammals.

## Materials and methods

### Mouse

#### Experimental animals

Wild type (WT) mice (*Mus musculus*, OF1 strain) were kept on a 12 h light/dark cycle with free access to food and water. Experiments were performed at embryonic (E), postnatal (P) and adult stages: four WT adult mice, one WT newborn, and three WT embryos were used in the present study. One adult male and two E16.5 GAD67-GFP knock-in embryos (Tamamaki et al., 2003), resulting of the insertion of the cDNA encoding EGFP into the locus encoding GAD67, have been used. After mating, the day of detection of the vaginal plug was considered as embryonic day E0.5. Pregnant females were sacrificed by decapitation at E14.5, E16.5, or E18.5. Uterine horns were removed from the mother and embryos were excised from their individual bag, soaked in cold 0.1 M Phosphate Buffer (PB) for 10 min and fixed by immersion in cold 2% paraformaldehyde (PFA) solution in 0.1 M PB for 3 h at 4°C. Newborn mice (P1) were deeply anesthetized by an exposure to low temperature and the spinal cord was immediately removed and fixed with 2% PFA for 4 h. Adult mice were deeply anesthetized with intraperitoneal injection of Nembutal® (150 mg·kg^−1^) and transcardially perfused sequentially with NaCl 0.9% (20 ml) and PFA (2%, 30 ml for WT adults and 4%, 30 ml for the GAD67-GFP mouse). After fixation, spinal cords were dissected out and post-fixed for 24 h in the fixative solution (2% PFA for WT mice and 4% for the GAD67-GFP mouse) at 4°C. Following fixation (and post-fixation if appropriate), tissues were cryo-protected for 24–48 h in 30% sucrose in 0.1 M PB at 4°C and then stored at -20°C for later use. Experiments were performed in accordance with the European Communities Council (EEC) Directive of 22 September 2010 (2010/63/EU) and French law (87/848).

#### Immunohistochemistry

Standard immunofluorescence procedures were used to localize antigens of interest (Voituron et al., 2011). At the cervical level, 10 μm-thick coronal sections of the spinal cord were obtained using a cryostat (Leica CM 3050S), mounted on silanized slides and stored at −20°C for later IHC using a rabbit anti-Polycystin-L (anti-human PKD2L1, 1:500 dilution; Millipore, Billerica, MA, USA). This PKD2L1 antibody was raised against the synthetic peptide from the N-terminal of human Polycystin-L. For PKD2L1 IHC at E14.5 and E16.5, we used an Antigen Unmasking Solution (Vector Laboratories). Briefly, the sections were incubated in 1% Bovine serum albumin (BSA) and 0.1% Triton X-100 for 20 min at room temperature (RT). After rinsing with 0.1 M phosphate buffered saline (PBS), the sections were incubated for 30 min at RT with DAPI (1.25 μg/ml; Life Technologies) and with Alexa-conjugated anti-rabbit secondary antibodies (1:500 dilution; Life Technologies). For GFP and PKD2L1 double IHC, 30 μm-thick coronal floating sections obtained using a cryostat (Leica CM 1850S) were immunostained by co-incubating two primary antibodies, a chicken anti-GFP (1:1000 dilution; Abcam, Cambridge, UK) with the rabbit anti-PKD2L1 (1:500 dilution) overnight at 4°C, and then the specific secondary antibodies Alexa Fluor 488 donkey anti-chicken IgG (1:500 dilution, Life Technologies) and Alexa 555-conjugated goat anti rabbit for 2 h at RT. In all IHC experiments, control sections were processed in parallel by omitting primary antibodies. Finally, sections were washed with PBS before being mounted onto glass slides with fluorescent mounting medium (AquaPolyMount, Biovalley, Marne La Vallée, France). Sections were observed under an Olympus FV1000 confocal microscope.

#### Quantification of cells

On 10 μm-thick sections, we performed Z-stacks with a step size of 1–1.5 μm in a region of interest (ROI) centered on the central canal (approximately 150 × 220 μm width, *n* = 13 sections for E16.5 GAD67-GFP and 115 × 140 μm width, *n* = 7 sections for adult GAD67-GFP sections). For each analyzed section, cells were identified by the DAPI staining in order to avoid counting multiple times the same cell. Within the ROI, all PKD2L1^+^ cells were counted manually and probed for GFP in the GAD67-GFP line.

### Macaque

#### Experimental animals

Tissue from two adult macaques (*Macaca fascicularis*, a 25-year old female and an 8-year old male) was used for this study. These animals were sacrificed for other purposes and spinal cord tissue samples were subsequently collected and devoted to the present study. Spinal cord collection was performed after the sacrifice of animals, in strict accordance with the recommendations of the Weatherall Report regarding good animal practice. All surgical procedures and experimental protocols were carried out in strict accordance with the National Institutes of Health guidelines (2013) and the recommendations of the EEC (2010/63/EU).

#### Tissue extraction and preparation

Macaques were deeply anesthetized with intramuscular injection of ketamine and xylazine at 50 and 5 mg/kg, respectively. Following the anesthesia, each animal was exsanguinated and perfused with 0.9% NaCl before their tissues were fixed by an intracardial perfusion containing 4% PFA in 0.1 M PBS. Spinal cord was extracted and meninges were removed using fine forceps. Tissue was embedded in 5% agarose and sectioned at 50 μm using a vibratome (Leica VT1000S). Additionally, frozen tissue blocks were obtained by passing tissue through a series of cryoprotection solutions (20, 30% sucrose in PBS) and freezing in isopentane; frozen sections were cut at 20 μm (HM 650V Microtome, Thermo Scientific). All sections were stored in 0.4% Sodium Azide in 0.1 M PBS at 4°C until IHC.

#### Immunohistochemistry

Free-floating sections from cervical, thoracic, and lumbar spinal cord tissue were used for IHC. Sections were first incubated in a blocking solution composed of 1% BSA, 0.5% Triton X-100 and 2% normal goat serum in PBS to reduce non-specific labeling. Sections were then incubated in primary antibodies overnight at 4°C. After multiple washes, sections were incubated in secondary antibodies for 4 h at 4°C. Primary antibodies used were rabbit anti-Polycystin-L (anti-human PKD2L1, 1:1,000 dilution; Millipore, Billerica, MA, USA), guinea pig anti-vesicular GABA Transporter (anti-VGAT, 1:500; Synaptic Systems, Germany) and rabbit anti-GAD65/67 (1:500; Abcam, MA, USA). Secondary antibodies were Alexa Fluor 488- or Alexa Fluor 568-conjugated anti-rabbit (1:500; Life Technologies) and Alexa Fluor 488-anti-guinea pig (1:500; Life Technologies). The same blocking solution as previously was used to dilute the antibodies. PBS was used for washing sections between each step. Sections were mounted on glass slides, cover slipped with Prolong® Gold Antifade Reagent (Life Technologies) mounting medium and then imaged on a Leica SP2 AOBS AOTF inverted confocal laser scanning microscope. Single optical slice images of individual labeled cells were taken using a 63× oil objective.

### Zebrafish

#### Experimental animals

All zebrafish (*Danio rerio*) lines were maintained and raised on a 14/10 h light cycle and water was regulated at 28.5°C, conductivity at 500 μS and pH at 7.4 (Westerfield, 2000). WT AB and Tüpfel long fin (TL) embryos were used for whole mount *in situ* hybridization (ISH). *Tg(nkx2.2a:mEGFP)* (Ng et al., 2005; Kirby et al., 2006) and *Tg(olig2:EGFP)* (Shin et al., 2003) transgenic lines were kindly provided by Prof. Bruce Appel, University of Colorado, Denver, USA. Embryos were dechorionated and staged according to number of somites as described (Kimmel et al., 1995): the 30-somite stage corresponds to Prim-5 or 24 h post fertilization when raised at 28.5°C. Adult fish were anesthetized in 0.02% MS 222 (Sandoz, Levallois-Perret, France) and killed by decapitation. All procedures were approved by the Institutional Ethics Committee at the Institut du Cerveau et de la Moelle épinière (ICM), Paris, France, the Ethical Committee Charles Darwin and received subsequent approval from the EEC (2010/63/EU).

#### Generation of the pkd2l1 probe

To generate the *pkd2l1* ISH probe, the coding fragment for *pkd2l1* was amplified from zebrafish embryo total cDNA using the following primers (5' to 3'): pkd2l1_For: TAGTGGTGATACTGCTTGCTGTGGTGG (from the end of the exon 6 of the *pkd2l1* gene and the beginning of exon 7) and pkd2l1_Rev: TGGTTCCACACTGTTCTCGAGGTCACG (from the end of exon 13). The PCR fragment was cloned into the pCRII-TOPO vector (Life Technologies, Carlsbad, CA, USA). The resulting plasmid was linearized with NotI. The *gad67* plasmid, kindly provided by Dr. Uwe Strähle, Karlsruhe Institute of Technology, Germany, was linearized with NcoI. Digoxigenin (DIG)- and fluorescein (Fluo)-labeled probes were synthesized using SP6 RNA polymerase with the RNA Labeling Kit (Roche Applied Science, Basel, Switzerland) to generate both *pkd2l1* and *gad67* antisense probes. To generate *pkd2l1* sense probes, the plasmid was linearized with KpnI and transcription was carried out using T7 RNA polymerase. All probes were purified using the mini Quick Spin RNA Column (Roche, Basel, Switzerland).

#### In situ hybridization

Whole-mount ISH were performed as previously described (Parmentier et al., 2011; Alunni et al., 2013) on embryos or dissected adult spinal cords fixed in 4% PFA in PBS overnight at 4°C. To reveal *pkd2l1* expression, probes were detected with anti-DIG or anti-Fluo antibodies conjugated to alkaline phosphatase followed by a chromogenic reaction using a solution of NBT/BCIP as substrate (Roche Diagnostics, France). To quantify cell density or ascertain transcript colocalization, probes were detected by the antibodies conjugated to horseradish peroxidase and were revealed by Tyramide Signal Amplification using Tyramide-FITC or Tyramide-TAMRA as substrates. The specificity of the *pkd2l1* probe was verified using a *pkd2l1* sense probe as negative control (data *not shown*). Double fluorescent *in situ* hybridization (FISH) for *pkd2l1* and *gad67* were performed on adult zebrafish spinal cords and coupled with respectively DIG and Fluo.

#### Immunohistochemistry and sectioning

The following primary antibodies were used for IHC: rabbit anti-GABA (1:2000, Sigma-Aldrich, St. Louis, MO, USA), rabbit anti-GAD65/67 and chicken anti-GFP (both used at 1:500 dilution, Abcam, Cambridge, UK). Immunostaining specificity was established by omitting the primary specific antibodies, no immunoreactive signal was observed. Agarose sections were collected as described above. In the zebrafish embryo, we performed either 50 μm-thick sagittal (for anti-GAD65/67 IHC) or transverse (for anti-GFP IHC) sections. In the adult, we performed 50 μm-thick sagittal and frontal sections of previously FISH stained adult spinal cords.

#### Combination of pkd2l1 FISH with immunohistochemistry

*pkd2l1* FISH was performed before IHC against GFP or GAD65/67: embryos were washed and immunostained with the chicken anti-GFP antibody or the rabbit anti-GAD65/67 antibody overnight at 4°C, and then incubated with the corresponding Alexa conjugated secondary antibodies IgG (1:500, Life Technologies) combined with DAPI (2.5 μg/ml, Life Technologies).

#### Microscopy

Whole-mount embryos stained by NBT/BCIP were mounted in 80% glycerol. Sections of adult spinal cord were mounted in a solution of Mowiol. Embryos were imaged using a Nikon AZ100M macroscope and a Leica DM5000 B Upright microscope. Adult spinal cord sections were imaged using a Nikon AZ100M macroscope. To quantify *pkd2l1*^+^ and GABA^+^ cells, stained embryos were imaged using the Fixed Stage microscope AxioExaminer Z1 equipped with a 20× water immersion objective and a Yokogawa CSU-X1 spinning disk unit (*n* = 13 embryos for *pkd2l1* and *n* = 10 for GABA). To quantify the overlap of GFP with *pkd2l1* FISH in the *Tg(nkx2.2a:mEGFP)* and the *Tg(olig2:EGFP)* transgenic embryos, 50 μm-thick sections were analyzed on an Olympus FV1000 confocal microscope equipped with a 40× water immersion objective using 405, 473, and 543 nm laser lines. Images were processed using Fiji (Schindelin et al., 2012) and Adobe Illustrator (Adobe Systems, Mountain View, CA, USA) software.

#### Quantification of cells

To quantify the total number of *pkd2l*1**^+^ cells revealed by FISH per embryo, Z-stacks of the entire embryos from somite 1 to 30 were acquired. Total numbers of cells were counted per somite labeled on both sides of the midline. The boundaries for each somite were established with transmitted light. The three subtypes of *pkd2l1* expressing cells were defined according to their position relative to the central canal. We distinguished: (i) the row of cells that was ventral and in proximity to the central canal, (ii) the row that was dorsal and in proximity to the central canal and (iii) the sparse cells that were dorsal and distant from the central canal. The proportion of *pkd2l1*^+^ cells double-labeled for GAD65/67 in 30-somite (Prim-5) embryos was quantified based on 50 μm-thick sagittal sections performed after the FISH and before GAD65/67 IHC. The proportion of *pkd2l1*^+^ cells double-labeled for GFP in *Tg(nkx2.2a:mEGFP)* and *Tg(olig2:EGFP)* in 30-somite embryos was quantified based on 50 μm-thick transverse sections performed after the FISH and IHC. The total number of cells is given as the mean ± Standard Error of the Mean (SEM). The same method was applied for counting of GABA^+^ KAs. There are four GABAergic interneuron types in the zebrafish embryo (Bernhardt et al., 1992; Higashijima et al., 2004a); KAs were identified as the only GABAergic ascending neurons with a soma located just below (KA”) or above (KA') the central canal.

## Results

### PKD2L1 is expressed in CSF-cNs from embryonic stages to adulthood in the mouse spinal cord

**Figure 1:**
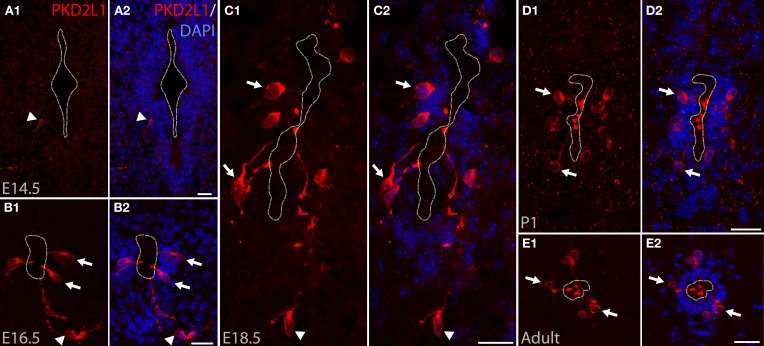
**PKD2L1 expression in CSF-cNs is present from embryonic stage E14.5 to adulthood in mouse. (A–E)** Immunostaining for PKD2L1 at E14.5 **(A)**, E16.5 **(B)**, E18.5 **(C)**, P1 **(D)**, and in the adult **(E)** performed on coronal sections of the mouse cervical spinal cord. At E14.5, few PKD2L1^+^ cells are detected (arrowhead). From E16.5 **(B)** to adult **(E)**, multiple PKD2L1^+^ CSF-cNs (arrows) surround the central canal where they project an apical bulbous dendritic extension contacting its lumen. Note that PKD2L1^+^ cells exhibiting a similar morphology but not distinctly contacting the central canal (arrowheads) can also be observed **(A–C)**. Dorsal is up. White dashed line delineates the central canal. DAPI staining appears in blue. Scale bars = 20 μm.

PKD2L1 was originally identified in CSF-cNs at postnatal stages P1–P4 in the mouse spinal cord (Huang et al., 2006). We investigated PKD2L1 expression in the spinal cord at the embryonic, postnatal and adult stages. PKD2L1 expression could not be detected before E14.5 in the spinal cord at the cervical, thoracic and lumbar levels. At E14.5, few PKD2L1^+^ cells could be identified in the cervical spinal cord (Figure 1A). Starting at E16.5, PKD2L1^+^ cells localized around the central canal exhibited the typical morphology of spinal CSF-cNs (Figures 1B–E, arrows). At E18.5, the soma of PKD2L1^+^ cells was usually located under the layer of ependymal cells (91 out of 102, while 11 out of 102 were located within the ependyma, Table 1). PKD2L1^+^ cells sent an apical bulbous extension toward the central canal ending in the lumen with a bud enriched in PKD2L1 (Figures 1B–E, arrows). In the adult, the soma of PKD2L1^+^ CSF-cNs was located in lamina X and always under the ependyma (95 out of 95 PKD2L1^+^ cells) (Figure 1E, Table 1). We also observed PKD2L1^+^ cells with similar morphology localized away from the central canal, though these cells lacked a visible apical extension to the central canal (Figures 1A–C, arrowheads). Our data identify PKD2L1 as a general marker of CSF-cNs.

**Table 1:** **Distribution of PKD2L1^+^ cells based on the location of their soma relative to the ependyma in the mouse spinal cord**.

**Stage**	**Total number of PKD2L1^+^ cells**	**Number of PKD2L1^+^ cells located in the ependymal layer**	**Number of PKD2L1^+^ cells located in the subependymal layer**
E16.5	84	8	76
E18.5	102	11	91
P1	184	6	178
Adult	95	0	95

PKD2L1^+^ cells located within the ependyma represent only a small number (<11%) of the total population of PKD2L1^+^ cells counted at embryonic and newborn stages. In the adult mouse spinal cord, the somata of all PKD2L1^+^ cells are located subependymally.

### PKD2L1 is present in GABAergic CSF-cNs in the mouse spinal cord

**Figure 2:**
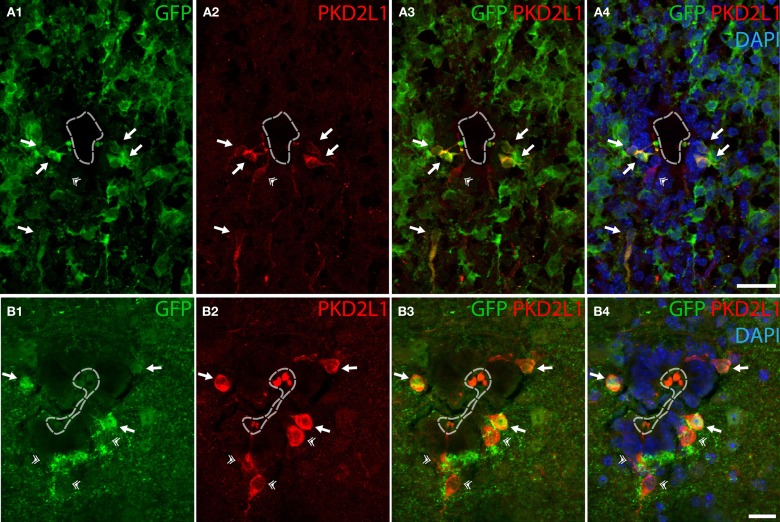
**Some PKD2L1^+^ cells surrounding the central canal of cervical spinal cord of adult mouse are immunoreactive for GFP in the knock-in GAD67-GFP mouse. (A)** 10 μm-thick coronal sections of GAD67-GFP E16.5 mouse spinal cord immunostained for GFP **(A1,A3,A4)** and PKD2L1 **(A2–A4)**. Most PKD2L1^+^ cells are GFP^+^ (arrows) but not all (double arrowheads). **(B)** 30 μm-thick coronal sections of a GAD67-GFP adult mouse spinal cord immunostained for GFP **(B1,B3,B4)** and PKD2L1 **(B2–B4)**. While many PKD2L1^+^cells are distinctly GFP^+^ (arrows), some cells are not (double arrowheads). Dorsal is up. White dashed line delineates the central canal. DAPI staining appears in blue. Scale bars = 25 μm **(A)**, 15 μm **(B)**.

As mentioned before, cells contacting the CSF had been described as GABAergic in many species, but the neurotransmitter phenotype has not been thoroughly established yet in mouse. We tested whether CSF-cNs were GABAergic in the embryonic and adult mouse spinal cord (Figure 2). We took advantage of the well-characterized GAD67-GFP knock-in mouse where the enhanced GFP was targeted to the locus encoding GAD67 using homologous recombination (Tamamaki et al., 2003). In these animals, we performed IHC for GFP to amplify the endogenous GFP signals (Figure 2). In GAD67-GFP knock-in embryos, double IHC for GFP and PKD2L1 on coronal sections of E16.5 mice spinal cords (Figure 2A) revealed that most of PKD2L1^+^ cells were GFP^+^ (arrows) indicating their GABAergic nature (*n* = 2 embryos, 107 out of 162 PKD2L1+ cells). In one adult, we observed the same findings and found 47 out of 73 cells labeled for both PKD2L1 and GFP (Figure 2B, arrows). Note that both in the embryos and in the adult, we observed PKD2L1^+^ cells that were not distinctly GFP^+^ (Figures 2A,B, double arrowhead). Notably, we never observed GFP^+^ CSF-cNs that were not PKD2L1^+^. Altogether we show evidence for PKD2L1^+^ CSF-cNs that are GABAergic in the mouse spinal cord.

### PKD2L1^+^ GABAergic CSF-cNs surround the central canal in the adult macaque spinal cord

**Figure 3:**
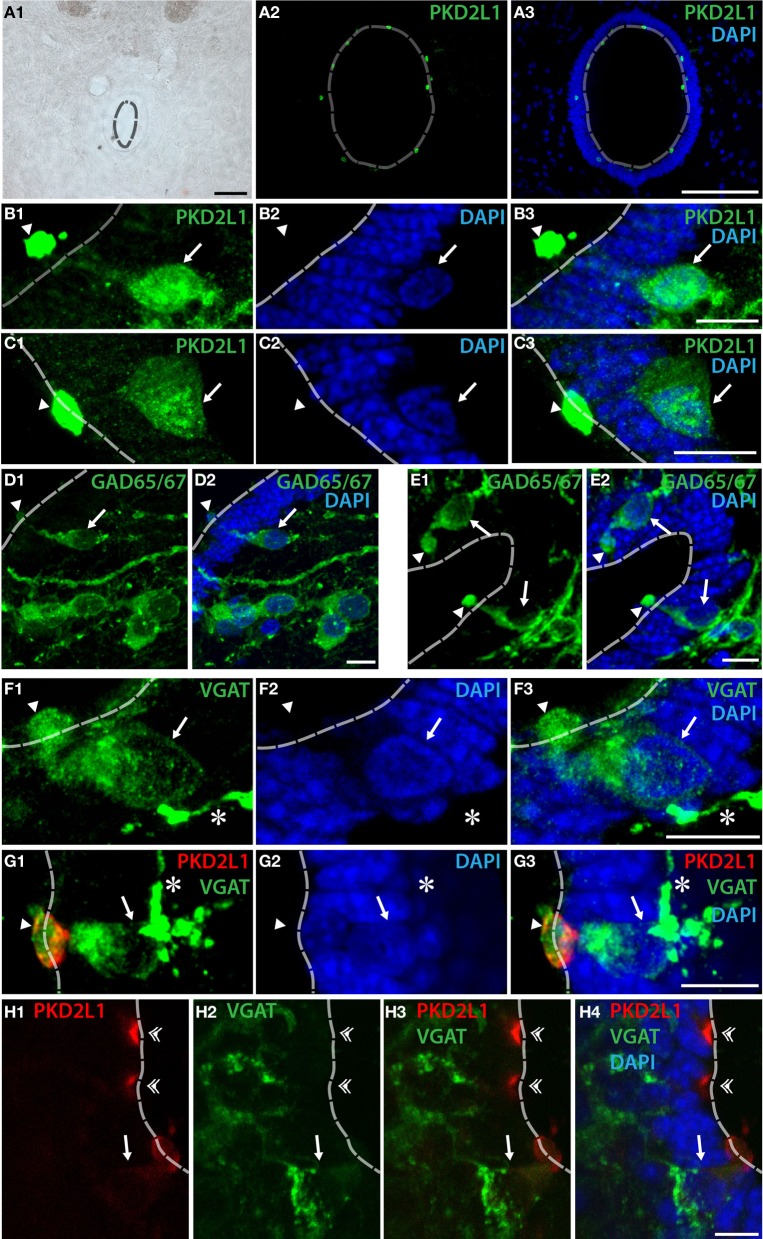
**PKD2L1^+^ CSF-cNs can be found as GABAergic neurons surrounding the central canal in the spinal cord of the adult macaque. (A–C)** PKD2L1 immunostaining labels intraluminal buds **(A–C)** and rarely subependymal or ependymal cell bodies **(B,C). (D,E)** GAD65/67 immunoreactive cells are numerous in the vicinity of the central canal: proximal cells show a clear apical extension contacting the lumen while cells located away do not. **(F)** CSF-cNs surrounding the central canal are immunoreactive for the vesicular GABA transporter VGAT. **(G,H)** Double immunostaining for VGAT and PKD2L1 shows that some but not all PKD2L1^+^ CSF-cNs are clearly GABAergic. The intraluminal buds can be found double-labeled for PKD2L1 and VGAT (arrowheads) but not systematically (double arrowheads), while apical extensions, cell body and putative axon are usually only labeled by VGAT. All panels are coronal sections of the macaque adult spinal cord taken at the lumbar level for **(A,G)**, at the cervical level for **(B–F)** and at the thoracic level for **(H)**. In all panels, arrows point to the somata, asterisks to the putative axons and arrowheads to the intraluminal buds of CSF-cNs. White dashed line delineates the central canal. DAPI staining appears in blue. Scale bars = 100 μm **(A)** and 10 μm **(B–H)**.

We next asked whether PKD2L1 labels CSF-cNs in the spinal cord of primates (Figure 3). In transverse sections from cervical, thoracic and lumbar spinal cord, we observed the distribution of PKD2L1 immunofluorescence around the central canal in lamina X (Figures 3A–C). PKD2L1^+^ cells were localized around the central canal and exhibited the typical morphology of spinal CSF-cNs with an apical extension toward the central canal ending with a bud in the lumen. PKD2L1 was enriched in the terminal bud of cerebrospinal fluid-contacting cells (Figures 3A–C) and faintly expressed (Figures 3B,C) or absent (Figure 3G) in the cell soma and in the rest of the apical extensions. PKD2L1^+^CSF-cNs closely surrounded the central canal; they had a round nucleus and were located under (Figures 3B–E) or within the ependyma (Figures 3F,G). In order to test whether PKD2L1^+^ CSF-cNs were GABAergic, we used IHC for the enzymes GAD65/67 and the VGAT transporter. Positive immunostaining for GAD65/67 and VGAT was found in cells surrounding the central canal at the level of their apical bulbous extension, soma and putative axon (Figures 3D–F, arrowheads, arrows, and asterisks, respectively) but buds were labeled by GABAergic markers less frequently than they were by PKD2L1. Double immunofluorescence for PKD2L1 and VGAT showed evidence for CSF-cNs double-labeled at the level of the soma and the intraluminal bud (Figure 3G). Nonetheless VGAT could not be detected in all PKD2L1^+^ buds (Figure 3H, double arrowheads). Cells double-labeled for PKD2L1 and VGAT were observed at the cervical, thoracic and lumbar level in the spinal cord. Taken together, our data show for the first time the presence of immunoreactive CSF-cNs containing PKD2L1 and GABAergic markers (GAD65/67 or VGAT) in the macaque lamina X. These findings demonstrate that a population of GABAergic CSF-cNs expressing PKD2L1 is present as well in the spinal cord of adult primates. Our results also support the idea that PKD2L1 is a specific marker of CSF-cNs in primates and labels more of these cells than GABAergic markers do in our experimental conditions.

### The expression of *pkd2l1* mRNA in zebrafish embryo enables an extensive quantification and lineage analysis of CSF-cNs

The developmental origin of CSF-cNs has not been established yet in mammals. To tackle the question of the developmental origin of *pkd2l1*^+^ cells, we took advantage of the zebrafish embryo for its transparency and the rapidity of development of the spinal cord.

#### pkd2l1 is expressed in CSF-cNs in zebrafish

**Figure 4:**
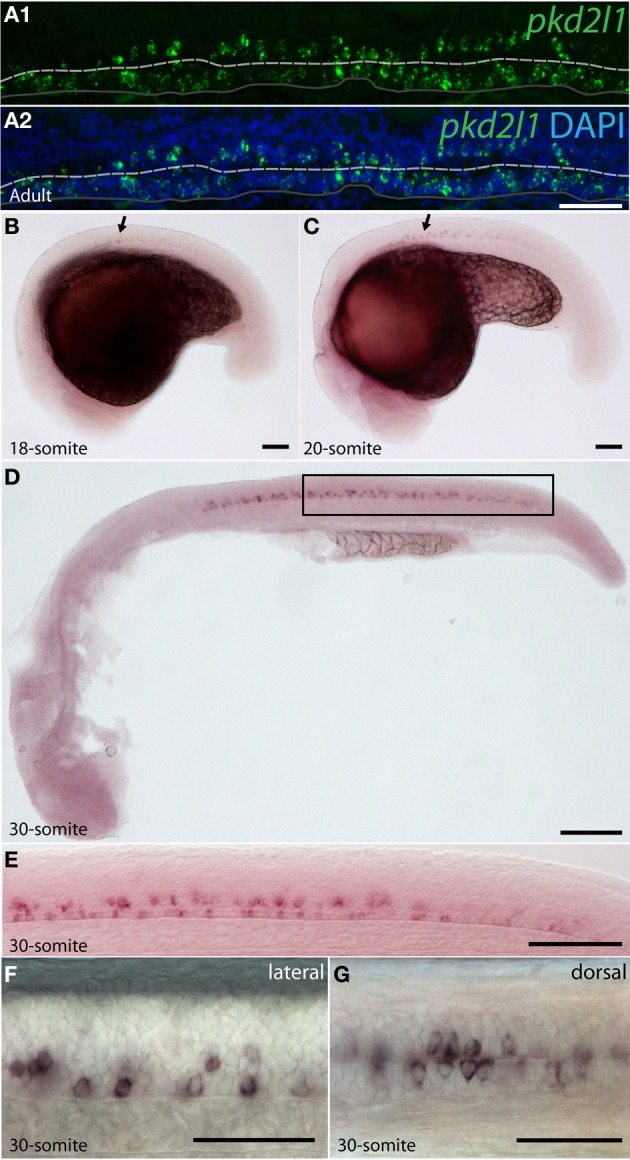
***pkd2l1* is expressed from the embryonic 18-somite stage to adulthood in the zebrafish spinal cord. (A)** Adult; **(B–G)** embryonic stages: 18-somite **(B)**, 20-somite **(C)** and 30-somite stages **(D–G)**. **(A)** In sagittal sections from the adult spinal cord, *pkd2l1*^+^ CSF-contacting cells are located ventral and dorsal to the central canal. White dash line: central canal, dark gray line: ventral limit of the spinal cord. *pkd2l1* expression appears in the rostral spinal cord (**B,C**, arrows) and is distributed in two rows of cells along the rostro-caudal axis **(E,F)** and on each side of the midline **(G)**. **(E)** is a close-up of the black box from **(D)**. Rostral is to the left for all panels. Lateral views with dorsal up for **(A–F)** and dorsal view for **(G)**. DAPI staining appears in blue. Scale bars = 40 μm **(A)**, 100 μm for **(B–D)**, 50 μm for **(E–G)**.

**Figure 5:**
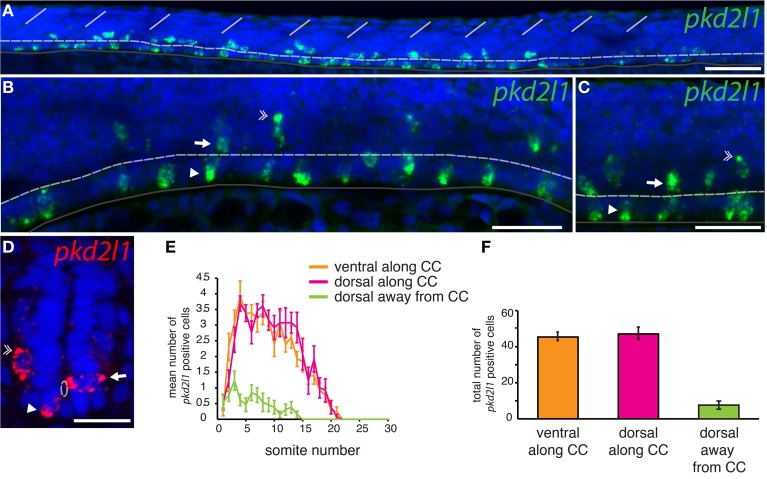
***pkd2l1* is expressed in cells contacting the CSF and surrounding the central canal in the spinal cord of the zebrafish embryo. (A–D)** FISH of *pkd2l1* mRNA at the 30-somite stage. Lateral views for **(A–C)** and transverse section for **(D)**. Two subpopulations of *pkd2l1* bright expressing cells surround the central canal ventrally (arrowhead) and dorsally (arrow). Note the existence of dorsal *pkd2l1* weak expressing cells away from the central canal (double arrowhead). The white dashed line, dark gray solid line and the solid white lines indicate respectively the central canal, the ventral limit of the spinal cord, and the somite boundaries. DAPI staining appears in blue. Scale bars = 50 μm for **(A)**, 30 μm for **(B,C)**, and 20 μm for **(D)**. **(E)** Mean number of *pkd2l1*^+^ cells per somite along the rostro-caudal axis at the 30-somite stage. **(F)** Total cell counts per embryo (*n* = 13 embryos). We counted 45.4 ± 2.4 ventral *pkd2l1*^+^ cells, 47.1 ± 3.4 dorsal *pkd2l1*^+^ cells and 7.6 ± 2.3 dorsal away from the central canal *pkd2l1*^+^ cells per embryo. **(E,F)** Mean values are given ± s.e.m.

To assess whether *pkd2l1* was expressed in CSF-cNs in zebrafish, we performed ISH using the antisense probe for *pkd2l1* (Figure 4). We observed the expression of *pkd2l1* in CSF-cNs along the central canal in sagittal sections of the adult spinal cord (Figure 4A). *pkd2l1*^+^ cells contacting the CSF were located ventrally and dorsally to the central canal (Figures 4A1,A2). In the zebrafish embryo, we investigated the 10-, 14-, 18-, 20-, and 30-somite stages (*n* > 6 embryos per condition). A weak signal in the rostral spinal cord (black arrow) could be detected at the 18-somite stage (Figure 4B). Gradually, the staining appeared rostro-caudally within the spinal cord (Figures 4C,D). Cells with dense labeling were located in two parallel rows along the ventral margin of the spinal cord (Figures 4E,F). Dorsal views of the spinal cord showed that these cells were located on either side of the midline (Figure 4G). At the larval stages, we observed that *pkd2l1*^+^ CSF-cNs covered the entire length of the spinal cord and the organization in two parallel rows was not distinct anymore (data *not shown*). In zebrafish, CSF-cNs (named KAs) have been shown to derive from two progenitor domains, p3 and pMN (Park et al., 2004; Schafer et al., 2007; Shin et al., 2007; Yeo and Chitnis, 2007; Yang et al., 2010; England et al., 2011; Huang et al., 2012). KAs were therefore divided into two subpopulations on this basis, dorsal KA' and ventral KA” (Park et al., 2004). To identify the nature of *pkd2l1* expressing cells, we performed FISH in 30-somite stage zebrafish embryos (i.e., Prim-5 stage or 24 h post fertilization, Figure 5). This staining confirmed that expression of *pkd2l1* mRNA was localized in the ventral spinal cord and mainly distributed in two rows of cells along the rostro-caudal axis (Figures 5A–C). At higher magnification, we observed that these cells were lining the central canal either ventrally (arrowhead) or dorsally (arrow) (Figures 5B–D). Transverse sections confirmed that the somata of these cells directly surround the central canal (Figure 5D). While most *pkd2l1*^+^ cells lined the central canal, we observed faint *pkd2l1* expressing cells away from the central canal in the dorsal spinal cord (Figures 5C,D, double arrowhead). We quantified the number of cells for each subpopulation based on lateral views originating from 13 embryos at the 30-somite stage (Figure 5E). *pkd2l1*^+^ cells localized mainly in the rostral two thirds of the spinal cord in 30-somite stage zebrafish embryos with 3–4 cells per somite for both ventral KA” and dorsal KA' between somites 5 and 15. At this stage, cell density decreased from somite 16 to 21 after which expression ceased (Figure 5E). We quantified the total number of *pkd2l1*^+^ cells for each subpopulation per embryo (Figure 5F). We found that *pkd2l1*^+^ cells from the ventral row reached 45.4 ± 2.4 cells (*n* = 13 embryos), similar to counts performed previously on KA” at the same stage (Huang et al., 2012). These observations on cell body location and number suggest that the ventral *pkd2l1*^+^ cells are KA” and dorsal ones are KA'.

#### All pkd2l1^+^ CSF-cNs are GABAergic in the zebrafish spinal cord

**Figure 6:**
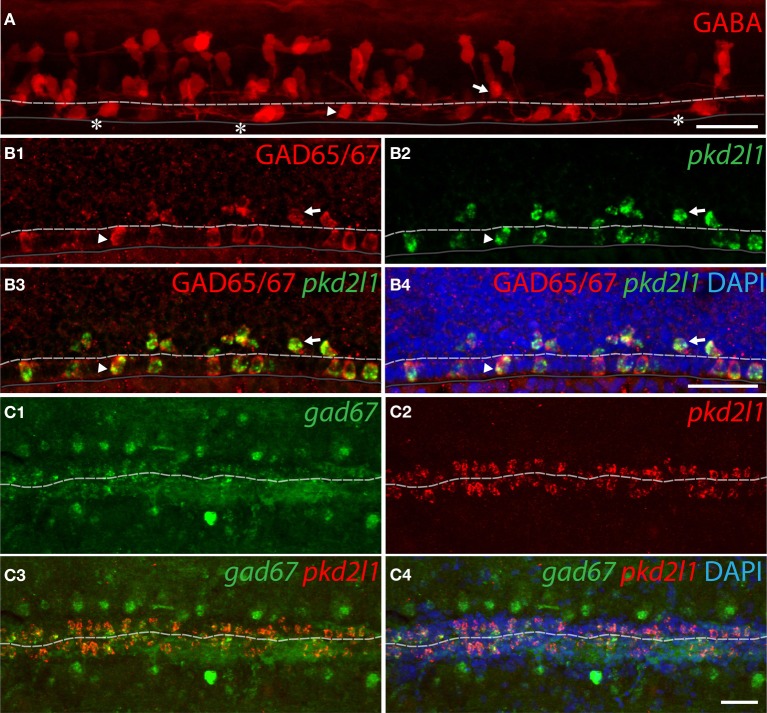
***pkd2l1*^+^ CSF-cNs are GABAergic neurons expressing GABA and GAD in zebrafish. (A)** GABA IHC on WT 30-somite embryos shows ventral (arrowhead) and dorsal (arrow) KAs and their ascending axon (asterisks). **(B1–B4)** Ventral and dorsal *pkd2l1^+^* KAs are GAD65/67 immunoreactive in 30-somite embryos as shown by FISH for *pkd2l1* (green) coupled to a GAD65/67 IHC (red). **(C1–C4)** In the adult, *pkd2l1*^+^ KAs are *gad67*^+^ as shown by FISH for *gad67* **(C1,C3,C4)** and *pkd2l1* **(C2–C4)** on sections of WT spinal cord. The white dash line indicates the central canal, the dark line the ventral limit of the spinal cord. **(A)** is a projection from the lateral view of a whole-mount embryo immunostained for GABA while **(B1–B4)** correspond to sagittal sections and **(C1–C4)** to frontal sections. DAPI staining appears in blue. Scale bars = 30 μm.

To demonstrate that zebrafish *pkd2l1*^+^ CSF-cNs are GABAergic, we investigated the expression of GABA and of GAD65/67 (Figure 6). An immunostaining for GABA at the 30-somite stage revealed multiple GABAergic cell types (Bernhardt et al., 1992; Higashijima et al., 2004a) including ventral and dorsal cells surrounding the central canal (Figure 6A, arrowhead and arrow, respectively). A short ascending axon originating from the soma of these cells can be observed (Figure 6A, asterisks). Based on morphology and location (see Materials and Methods), we estimated the number of GABAergic ventral (34.6 ± 2.3) and dorsal (49.9 ± 1.8) CSF-cNs per embryo (*n* = 10 embryos). We performed *pkd2l1* FISH followed by IHC for GAD65/67 to determine whether *pkd2l1*^+^ CSF-cNs are also GABAergic. All *pkd2l1*^+^ cells (*n* = 160) along the central canal were GAD65/67 immunoreactive (Figure 6B) (92 ventral *pkd2l1*^+^ cells, 68 dorsal *pkd2l1*^+^ cells). Based on their location, density and GABAergic phenotype, these *pkd2l1*^+^ CSF-cNs correspond to KA” and KA'. To test whether zebrafish CSF-cNs persist as GABAergic neurons until adulthood, we performed *pkd2l1* and *gad67* double FISH on whole adult spinal cord indicating that *pkd2l1*^+^ CSF-cNs express *gad67* throughout development (Figure 6C). Contrary to what we observed in mouse and macaque, these results demonstrate that in zebrafish all *pkd2l1*^+^ CSF-cNs are clearly GABAergic.

#### pkd2l1^+^CSF-cNs originate from the p3 and pMN progenitor domains in zebrafish

**Figure 7:**
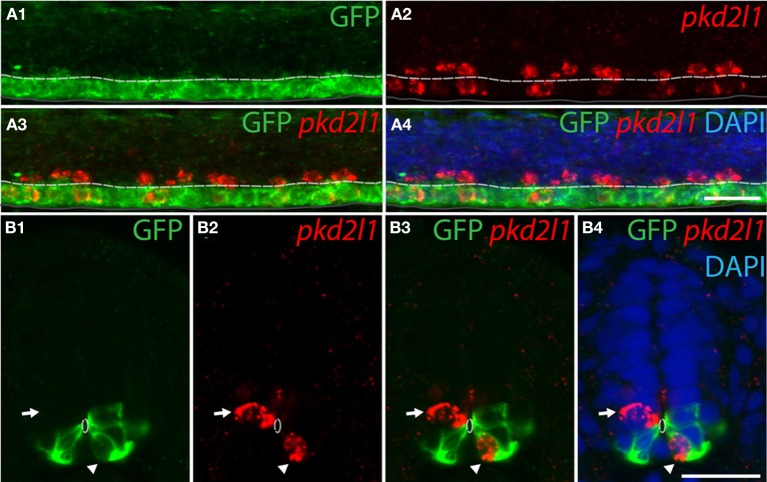
**Ventral *pkd2l1*^+^ cells express *nkx2.2a* and derive from the p3 progenitor domain in the zebrafish embryo. (A,B)** *pkd2l1* FISH in *Tg(nkx2.2a: mEGFP)* embryos immunostained for GFP at the 30-somite stage. IHC for GFP reveals the p3 domain. **(A)** Lateral view showing that the most ventral *pkd2l1*^+^ cells are GFP^+^. **(B)** A typical transverse section shows a ventral *pkd2l1*^+^ cell contacting the central canal and expressing GFP (arrowhead) while a dorsal *pkd2l1*^+^ cell contacting the central canal does not express GFP (arrow) in the *Tg(nkx2.2a: mEGFP)* transgenic embryo. White dashed line delineates the central canal, dark line the ventral limit of the spinal cord. DAPI staining appears in blue. Scale bars = 20 μm.

**Figure 8:**
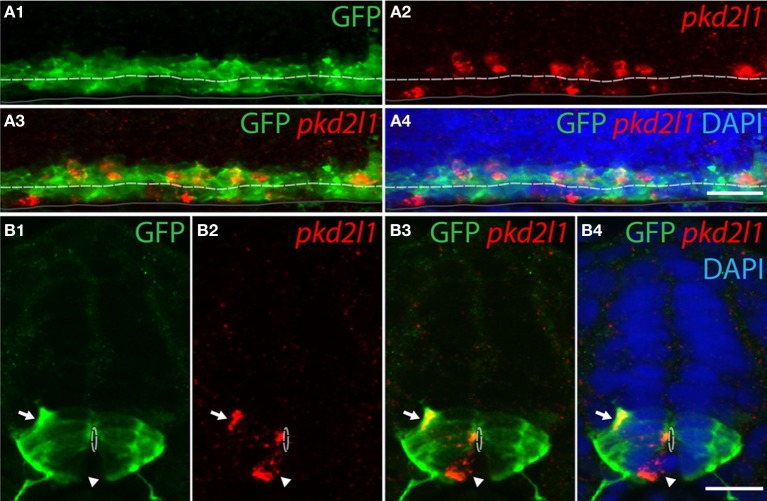
**Dorsal *pkd2l1*^+^ CSF-cNs express *olig2* and derive from the pMN progenitor domain in the embryonic spinal cord of zebrafish. (A,B)** *pkd2l1* FISH in *Tg(olig2:EGFP)* embryos immunostained for GFP at the 30-somite stage. IHC for GFP reveals the pMN domain. **(A)** Lateral view showing dorsal *pkd2l1*^+^ cells express GFP. **(B)** Transverse sections showing dorsal *pkd2l1*^+^ cells contacting the central canal express GFP (arrow) while a ventral *pkd2l1*^+^ cell contacting the central canal (arrowhead) does not express GFP in the *Tg(olig2:EGFP)* transgenic line. White dashed line delineates the central canal, dark line the ventral limit of the spinal cord. DAPI staining appears in blue. Scale bars = 20 μm.

To confirm the developmental origins of *pkd2l1*^+^ cells, we took advantage of the zebrafish model in which *pkd2l1* is expressed in the 30-somite stage embryo when progenitor domains are well-defined. Previous studies have shown that dorsal KA' cells are derived from the pMN domain and express the transcription factor *olig2* (Park et al., 2004). Ventral KA” cells originate from the p3 progenitor domain (Schafer et al., 2007) and express the transcription factor *nkx2.2a* (Yang et al., 2010; Huang et al., 2012). We therefore performed *pkd2l1* FISH combined with an anti-GFP immunostaining in the *Tg(nkx2.2a:mEGFP)* and the *Tg(olig2:EGFP)* transgenic lines (Shin et al., 2003; Ng et al., 2005) (Figures 7, 8). In the *Tg(nkx2.2a:mEGFP)* transgenic embryos, the p3 domain was labeled by GFP staining as expected (Figure 7A). Ventral *pkd2l1*^+^ cells contacting the central canal were labeled for GFP while dorsal cells were not (Figure 7B). In the *Tg(olig2:EGFP)* transgenic embryos (Figure 8), the progenitor domain pMN was labeled by GFP (Figure 8A). In these animals, only dorsal *pkd2l1*^+^ cells contacting the central canal were GFP^+^ (Figure 8B). Transverse sections allowed to estimate the number of *pkd2l1*^+^ cells contacting the central canal that were positive for GFP in the *Tg(nkx2.2a:mEGFP)* and in the *Tg(olig2:EGFP)* transgenic embryos (Table 2). While only ventral *pkd2l1*^+^ cells expressed GFP in the *Tg(nkx2.2a:mEGFP)* line (38 out of 38 cells), dorsal cells solely expressed GFP in the *Tg(olig2:EGFP)* line (93 out of 93 cells) (Table 2). Our data indicate that *pkd2l1*^+^ cells lining the central canal in zebrafish are KAs originating from p3 (for KA”) and from pMN (for KA') depending on their dorso-ventral location relative to the central canal.

**Table 2:** **Distribution of ventral and dorsal *pkd2l1*^+^ cells expressing GFP in *Tg(nkx2.2a:mEGFP)* and *Tg(olig2:EGFP)* zebrafish transgenic lines at the 30-somite stage**.

	**Number of cells**	**%**
**COLOCALIZATION OF GFP AND *pkd2l1* IN *Tg(nkx2.2a:mEGFP)***		
Total number of *pkd2l1*^+^ counted cells	76	
Ventral to CC *pkd2l1*^+^ GFP^+^/ventral to the CC *pkd2l1*^+^	38/38	100
Dorsal to CC *pkd2l1*^+^ GFP^+^/dorsal to the CC *pkd2l1*^+^	0/32	0
Dorsal and away from the CC *pkd2l1*^+^ GFP^+^/ dorsal and away from the CC *pkd2l1*^+^	0/6	0
**COLOCALIZATION OF GFP AND *pkd2l1* IN *Tg(olig2:EGFP)***		
Total number of *pkd2l1*^+^ counted cells	149	
Ventral to CC *pkd2l1*^+^ GFP^+^/ ventral to the CC *pkd2l1*^+^	0/52	0
Dorsal to CC *pkd2l1*^+^ GFP^+^/ dorsal to the CC *pkd2l1*^+^	93/93	100
Dorsal and away from the CC *pkd2l1*^+^ GFP^+^/ dorsal and away from the CC *pkd2l1*^+^	2/4	50

All ventral pkd2l1^+^ cells are GFP^+^ in Tg(nkx2.2a:mEGFP) but none of the dorsal ones. Reciprocally all the dorsal pkd2l1^+^ cells are GFP ^+^ in the Tg(olig2:EGFP) but none of the ventral ones.

## Discussion

### PKD2L1 appears as a specific marker of spinal CSF-cNs in bony vertebrate species

PKD2L1^+^ neurons projecting their apical bulbous dendritic extension into the central canal of the spinal cord had been described only in mouse at the P1–P4 stages (Huang et al., 2006) and in the adult (Orts-Del'Immagine et al., 2012, 2014). We hypothesized that the channel PKD2L1 could be a specific marker of spinal CSF-cNs shared among vertebrates. We demonstrate here that the TRP channel PKD2L1 is a marker of CSF-cNs in three vertebrate species: *Mus musculus*, *Macaca fascicularis*, and *Danio rerio*. For the first time, we show the expression of this channel in primate and zebrafish CSF-cNs. In the adult macaque spinal cord, CSF-cNs express PKD2L1 at the cervical, thoracic and lumbar levels. In the zebrafish embryo, *pkd2l1*^+^ CSF-cNs originally differentiate in two rows along rostro-caudal gradient.

Taken together, our results demonstrate the shared expression of PKD2L1 in spinal CSF-cNs across bony vertebrate species. GABA has been previously reported to label some CSF-cNs (Barber et al., 1982; Dale et al., 1987a,b; Brodin et al., 1990; Christenson et al., 1991a,b; Bernhardt et al., 1992; Martin et al., 1998; Binor and Heathcote, 2001; Stoeckel et al., 2003; Robertson et al., 2007; Rodicio et al., 2008; Villar-Cervino et al., 2008; Reali et al., 2011). In mouse and macaque, PKD2L1 appeared always highly enriched in the intraluminal buds of CSF-cNs. On the contrary, GABAergic markers (GAD65/67, VGAT) rarely labeled in these species the soma or the apical bulbous extension of CSF-cNs. Notably we never observed CSF-cNs labeled by GABAergic markers and not by PKD2L1. In mammals, PKD2L1 seems therefore to label more CSF-contacting cells than GABAergic markers do. Nonetheless, with the approach we developed here, we cannot assess the existence of CSF-cNs that would not express PKD2L1.

### Expression of PKD2L1 in CSF-cNs of the embryonic spinal cord

In mouse and zebrafish, we detected the expression of the channel at early embryonic stages of development (E14.5 stage for the mouse and 18-somite stage for the zebrafish). In mouse, PKD2L1 expression could be found in few spinal cells contacting the central canal at E14.5 but became more evenly expressed from E16.5 to adulthood. This observation is consistent with a recent report showing that CSF-cNs emerged at E14 in the rat spinal cord (Kutna et al., 2013). It suggests that PKD2L1 expression may start soon after the cell differentiates into a CSF-cN.

We demonstrate here that within the spinal cord of the zebrafish embryo *pkd2l1* mRNA is enriched in CSF-cNs that are mainly arranged in two rows, one ventral and one dorsal to the central canal. Previous studies have shown that KA cells can be subdivided into two populations of CSF-cNs: the ventral KA” and the dorsal KA' (Park et al., 2004; Schafer et al., 2007; Shin et al., 2007; Yeo and Chitnis, 2007; Yang et al., 2010; England et al., 2011; Huang et al., 2012). We showed that *pkd2l1* can be detected in both KA” and KA'.

In the three species studied here, we observed PKD2L1^+^ perikarya away from the central canal and for which we could not observe an apical bulbous extension reaching the lumen. In the zebrafish embryonic spinal cord, *pkd2l1* expression was weak in cells that were localized dorsally and away from the central canal. These cells were less than 8% (7.6% ± 2.3) of the *pkd2l1*^+^ cells in our FISH experiments (Figure 5). Some of them expressed GFP in the *Tg(olig2:EGFP)* line (Table 2) suggesting that they could be either CSF-cNs originating from pMN, motoneurons or ventral longitudinal descending neurons (VeLDs) (Bernhardt et al., 1990; Park et al., 2004; Warp et al., 2012). A more extensive characterization would be necessary to identify the nature of these marginal dorsal cells. In cross sections of mouse, macaque and zebrafish spinal cords, those distant cells did not distinctly contact the central canal. However, as shown in rats (Lu et al., 2008), they could extend a long apical bulbous extension reaching the central canal that would be difficult to capture in thin sections.

### On the developmental origins of *pkd2l1* expressing CSF-cNs

At the embryonic stage, the neural tube is subdivided into molecularly defined neural progenitor domains generating distinct neuronal subtypes in vertebrates (Ericson et al., 1997; Briscoe et al., 1999; Jessell, 2000; Novitch et al., 2001; Goulding, 2009). In zebrafish, CSF-cNs referred to as KA cells derive from the two most ventral domains of the spinal cord; the ventral to the central canal p3 domain labeled by *nkx2.2a* and the more dorsal pMN marked by *olig2* (Park et al., 2004; Schafer et al., 2007; Shin et al., 2007; Yeo and Chitnis, 2007; Yang et al., 2010; England et al., 2011; Huang et al., 2012). By analyzing transverse sections of stable transgenic lines where GFP reports the expression of these transcription factors, we found that *pkd2l1*^+^ CSF-cNs were derived from the *nkx2.2a* and from the *olig2* expression domains. This observation confirms the double developmental origin of CSF-cNs in zebrafish.

In the embryonic mouse spinal cord, the progenitor domains p3 and pMN form well-defined bands labeled by NKX2.2 and OLIG2, respectively, between E9.5 and E12.5 (Briscoe et al., 1999; Jessell, 2000; Novitch et al., 2001). At these stages, PKD2L1 is not yet expressed (*data not shown*). Therefore, by IHC for these transcription factors and PKD2L1, we could not test whether PKD2L1^+^CSF-cNs originate as well from p3 and pMN (*data not shown*). To reveal the developmental origin of these cells in mouse, lineage tracing based on the use of inducible transgenic lines for p3 and pMN markers will be necessary. Previous results relying on a tamoxifen-inducible Cre-recombinase inserted into the Olig2 locus indicate that a subpopulation of cells originating from pMN between E9.5 and E14.5 are located at the ependymal border circling the central canal (Srinivas et al., 2001; Masahira et al., 2006). This observation suggests that some CSF-cNs could originate from pMN in mouse, although a thorough investigation would be necessary to address this question in this model organism.

### GABAergic CSF-cNs express PKD2L1 in the spinal cord

GABAergic CSF-cNs have been described in the spinal cord of various vertebrate species (Barber et al., 1982; Dale et al., 1987a,b; Brodin et al., 1990; Christenson et al., 1991a,b; Bernhardt et al., 1992; Martin et al., 1998; Binor and Heathcote, 2001; Stoeckel et al., 2003; Robertson et al., 2007; Rodicio et al., 2008; Villar-Cervino et al., 2008; Reali et al., 2011). Here we used different GABAergic markers, GABA itself, the synthesis enzyme GAD or the GABA transporter VGAT, to test whether PKD2L1^+^ CSF-cNs were GABAergic. In zebrafish, we demonstrate that all *pkd2l1*^+^ cells are GABAergic in spinal CSF-cNs. In mouse and macaque, we could not demonstrate a complete co-localization of PKD2L1 with GABAergic markers (GAD67-GFP or VGAT) in CSF-cNs. In macaque, a minority of PKD2L1^+^ intraluminal buds was clearly double-labeled with VGAT. In mouse, the observation of GABAergic PKD2L1^+^ CSF-cNs was confirmed in the GAD67-GFP transgenic line by the co-expression of PKD2L1 and GFP in only some cells. The lack of systematic colocalization between PKD2L1 and GABAergic markers in CSF-cNs could be due to the expression of other isoforms of GAD (such as GAD65) or to other neurotransmitters expressed in PKD2L1^+^ CSF-cNs. Although we could not demonstrate that GABA is expressed in all CSF-cNs in mammals, our results show evidence for GABAergic PKD2L1^+^ CSF-cNs in the spinal cord of the three species studied here.

Previous studies characterized a diversity of markers potentially expressed in CSF-cNs in multiple species. Peptides such as the vasoactive intestinal polypeptide (VIP), somatostatin or urotensin II-related peptide (URP2) have been found in CSF-cNs (Buchanan et al., 1987; Yulis and Lederis, 1988a,b; Christenson et al., 1991a; Lamotte and Shapiro, 1991; Lopez et al., 2007; Wyart et al., 2009; Parmentier et al., 2011; Jalalvand et al., 2014). Ventral CSF-cNs were found dopaminergic in tetrapods such as birds (Acerbo et al., 2003) and amphibians (Gonzalez and Smeets, 1991, 1993; Gonzalez et al., 1993), as well as in some teleosts such as the eel and the trout (Roberts et al., 1995), in dogfish (Sueiro et al., 2004) and lampreys (Schotland et al., 1996; Rodicio et al., 2008). It would be interesting to determine in these species whether the dopaminergic and non-dopaminergic CSF-cNs derive as well from two different progenitor domains. However, the dopaminergic phenotype does not seem to be conserved in all vertebrate species as shown in some teleosts and in mammals (Nagatsu et al., 1988; McLean and Fetcho, 2004).

Other markers such as the subunit P2X_2_ of the purinergic receptor have been found in adult CSF-cNs together with early neuronal markers such as the polysialylated neural cell adhesion molecule (PSA-NCAM) in rats (Stoeckel et al., 2003) and HuC in turtles (Reali et al., 2011) and dogfish (Sueiro et al., 2004).

All markers listed above are not highly specific for CSF-cNs. However, in the three species studies here, PKD2L1 appears as a specific marker of CSF-cNs that is highly expressed and broadly targets CSF-cNs within the spinal cord. Despite existing variations in expression of peptides, neuromodulators and receptors, our results in zebrafish, mouse and macaque suggest the existence of a conserved system of spinal CSF-cNs defined by their morphology, their location—ependymally or subependymally—and their enriched expression of PKD2L1.

PKD2L1 has been involved in multiple functions from sour taste (Huang et al., 2006; Ishimaru et al., 2006; Inada et al., 2008; Ishii et al., 2009; Shimizu et al., 2009; Chang et al., 2010; Shimizu et al., 2011; Horio et al., 2011) to primary cilium signaling (Decaen et al., 2013; Delling et al., 2013). The peculiar location of CSF-cNs in contact with the CSF strongly suggests a role for PKD2L1 as a sensor of CSF composition, pH and/or osmolarity (Huang et al., 2006; Orts-Del'Immagine et al., 2012) since the channel is activated upon acidification (Ishimaru et al., 2006; Inada et al., 2008; Orts-Del'Immagine et al., 2012), alkalinization (Shimizu et al., 2011; Orts-Del'Immagine et al., 2012) or hypo-osmotic variations (Shimizu et al., 2009; Orts-Del'Immagine et al., 2012).

The role(s) of CSF-cNs in the vertebrate spinal cord is (are) poorly understood. They could be proprioceptors sensitive to the CSF composition (Huang et al., 2006; Orts-Del'Immagine et al., 2012) and modulating locomotion (Wyart et al., 2009), or enabling the differentiation of progenitors in the ependymal neurogenic niche via GABA release (Reali et al., 2011). The investigation of PKD2L1 functions in CSF-cNs across multiple species should reveal whether its physiological role in the spinal cord is conserved in vertebrates.

## Author contributions

Lydia Djenoune and Claire Wyart conceived, designed, and supervised all experiments. Lydia Djenoune performed the experiments on zebrafish embryos helped by Céline Burcklé at the early stages of the project. Hanen Khabou performed all experiments on the macaque spinal cord. Fanny Joubert and Laurence Bodineau performed experiments on the mouse spinal cord. Feng B. Quan performed FISH experiments on the zebrafish adult spinal cord. Sophie Nunes Figueiredo performed GABA immunohistochemistry on zebrafish embryos. Filippo Del Bene designed the *pkd2l1 in situ* probe. Lydia Djenoune analyzed the data under supervision of Claire Wyart and Hervé Tostivint. Lydia Djenoune and Claire Wyart wrote the manuscript. All authors discussed the results and implications and commented on the manuscript.

### Conflict of interest statement

The authors declare that the research was conducted in the absence of any commercial or financial relationships that could be construed as a potential conflict of interest.

## Abbreviations

cc: Central canal
CSF: Cerebrospinal fluid
CSF-cNs: Cerebrospinal fluid-contacting neurons
dpf: days post fertilization
FISH: Fluorescent *in situ* hybridization
GAD: Glutamic Acid Decarboxylase
GABA: γ-aminobutyric acid
GFP: Green Fluorescent Protein
GFP^+^: GFP positive
hpf: hours post fertilization
IHC: Immunohistochemistry
KAs: Kolmer–Agduhr cells
MAP2: microtubule-associated protein 2
PB: Phosphate Buffer
PBS: Phosphate Buffered Saline
PFA: paraformaldehyde
*pkd2l1*: mRNA form of polycystic kidney disease 2-like 1 in zebrafish
*pkd2l1*^+^: *pkd2l1* positive
PKD2L1: polycystic kidney disease 2-like 1 protein in mouse and macaque
PKD2L1^+^: PKD2L1 positive
TRP: Transient Receptor Potential
VGAT: vesicular GABA transporter.
